# First 100 Persons with COVID-19 — Zambia, March 18–April 28, 2020

**DOI:** 10.15585/mmwr.mm6942a5

**Published:** 2020-10-23

**Authors:** Peter J. Chipimo, Danielle T. Barradas, Nkomba Kayeyi, Paul M. Zulu, Kapina Muzala, Mazyanga L. Mazaba, Raymond Hamoonga, Kunda Musonda, Mwaka Monze, Nathan Kapata, Nyambe Sinyange, Davie Simwaba, Fred Kapaya, Lloyd Mulenga, Duncan Chanda, Warren Malambo, William Ngosa, Jonas Hines, Samuel Yingst, Simon Agolory, Victor Mukonka

**Affiliations:** ^1^Zambia National Public Health Institute, Lusaka, Zambia; ^2^Division of Global HIV and Tuberculosis, Center for Global Health, CDC; ^3^University Teaching Hospital Virology Laboratory, Lusaka, Zambia; ^4^University Teaching Hospital Adult Infectious Disease Center of Excellence, Lusaka, Zambia; ^5^Zambia Ministry of Health, Lusaka, Zambia.

Zambia is a landlocked, lower-middle income country in southern Africa, with a population of 17 million (*1*). The first known cases of coronavirus disease 2019 (COVID-19) in Zambia occurred in a married couple who had traveled to France and were subject to port-of-entry surveillance and subsequent remote monitoring of travelers with a history of international travel for 14 days after arrival. They were identified as having suspected cases on March 18, 2020, and tested for COVID-19 after developing respiratory symptoms during the 14-day monitoring period. In March 2020, the Zambia National Public Health Institute (ZNPHI) defined a suspected case of COVID-19 as 1) an acute respiratory illness in a person with a history of international travel during the 14 days preceding symptom onset; or 2) acute respiratory illness in a person with a history of contact with a person with laboratory-confirmed COVID-19 in the 14 days preceding symptom onset; or 3) severe acute respiratory illness requiring hospitalization; or 4) being a household or close contact of a patient with laboratory-confirmed COVID-19. This definition was adapted from World Health Organization (WHO) interim guidance issued March 20, 2020, on global surveillance for COVID-19 (*2*) to also include asymptomatic contacts of persons with confirmed COVID-19. Persons with suspected COVID-19 were identified through various mechanisms, including port-of-entry surveillance, contact tracing, health care worker (HCW) testing, facility-based inpatient screening, community-based screening, and calls from the public into a national hotline administered by the Disaster Management and Mitigation Unit and ZNPHI. Port-of-entry surveillance included an arrival screen consisting of a temperature scan, report of symptoms during the preceding 14 days, and collection of a history of travel and contact with persons with confirmed COVID-19 in the 14 days before arrival in Zambia, followed by daily remote telephone monitoring for 14 days. Travelers were tested for SARS-CoV-2, the virus that causes COVID-19, if they were symptomatic upon arrival or developed symptoms during the 14-day monitoring period. Persons with suspected COVID-19 were tested as soon as possible after evaluation for respiratory symptoms or within 7 days of last known exposure (i.e., travel or contact with a confirmed case). All COVID-19 diagnoses were confirmed using real-time reverse transcription–polymerase chain reaction (RT-PCR) testing (SARS-CoV-2 Nucleic Acid Detection Kit, Maccura) of nasopharyngeal specimens; all patients with confirmed COVID-19 were admitted into institutional isolation at the time of laboratory confirmation, which was generally within 36 hours. COVID-19 patients were deemed recovered and released from isolation after two consecutive PCR-negative test results ≥24 hours apart. A Ministry of Health memorandum was released on April 13, 2020, mandating testing in public facilities of 1) all persons admitted to medical and pediatric wards regardless of symptoms; 2) all patients being admitted to surgical and obstetric wards, regardless of symptoms; 3) any outpatient with fever, cough, or shortness of breath; and 4) any facility or community death in a person with respiratory symptoms, and 5) biweekly screening of all HCWs in isolation centers and health facilities where persons with COVID-19 had been evaluated. This report describes the first 100 COVID-19 cases reported in Zambia, during March 18–April 28, 2020.

These 100 positive test results were reported from 6,165 tests conducted during this time (1.6% positive); most (77%) of the 100 persons with COVID-19 were identified in the capital of Lusaka. Most cases occurred in men (61%) and in adults aged 30–44 years (32%) and were identified through point-of-entry surveillance (35%) and contact tracing (30%). Thirty-five persons with COVID-19 had traveled internationally in the 14 days before testing; 65 persons with locally acquired COVID-19 included 30 non-HCW contacts of a person with known COVID-19. Fever, cough, sore throat, headache, and fatigue were the most commonly reported signs and symptoms; 79% of cases were asymptomatic at the time of testing. Median recovery time was 12 days (interquartile range = 1–42 days) from date of symptom onset (or date of testing for asymptomatic patients). Underlying health conditions were reported by 20% of patients; among patients with underlying conditions, human immunodeficiency virus infection (35%) and hypertension (35%) were those most commonly reported. Three deaths were recorded; two of the patients who died received critical or intensive care before death, and all three had at least one underlying health condition.

During the first 28 days after confirmation of Zambia’s first COVID-19 case, 65% of cases were identified via point-of-entry surveillance and contact tracing (Figure). However, testing asymptomatic persons, including HCWs, in hospital settings where persons with confirmed COVID-19 were being cared for was helpful in identifying COVID-19 among 16 HCWs and four admitted patients and might have reduced nosocomial transmission.

**FIGURE:**
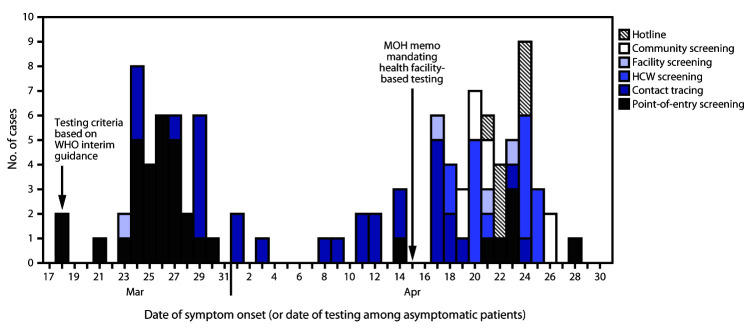
Mode of detection*^,†^ of first 100 confirmed COVID-19 cases — Zambia, March 18–April 28, 2020 **Abbreviations:** COVID-19 = coronavirus disease 2019; HCW = health care worker; MOH = Ministry of Health; WHO = World Health Organization. * WHO Interim Guidance: Global Surveillance for COVID-19 Caused by Human Infection with COVID-19 Virus. March 20, 2020. https://apps.who.int/iris/rest/bitstreams/1272502/retrieve. Zambia suspected case definition: 1) an acute respiratory illness in a person with a history of international travel during the 14 days preceding symptom onset; 2) acute respiratory illness in a person with a history of contact with a person with laboratory-confirmed COVID-19 in the 14 days preceding symptom onset; 3) severe acute respiratory illness requiring hospitalization; or 4) being a household or close contact of a patient with laboratory-confirmed COVID-19. ^†^ MOH memo released on April 13, 2020, mandated testing of 1) all persons admitted to medical and pediatric wards regardless of symptoms; 2) all patients before admission to surgical and obstetric wards, regardless of symptoms; 3) any outpatient with fever, cough, or shortness of breath; and 4) any facility or community death in a person with respiratory symptoms, and facility-based screening of HCWs.

After the first persons with COVID-19 with no apparent epidemiologic links to other reported persons with COVID-19 were confirmed in early April, the number of cases identified through community-based screenings in residential areas and nearby markets where the unlinked cases had been identified increased. Expansion of the national testing strategy to include asymptomatic persons with possible COVID-19 exposures and those with no international travel history facilitated detection and isolation of cases that would have been otherwise missed. Other countries in the region or with similar demographic profiles might find these strategies useful for detection, containment, or mitigation of COVID-19.
